# Selection signature analysis reveals genes underlying sheep milking performance

**DOI:** 10.5194/aab-62-501-2019

**Published:** 2019-08-08

**Authors:** Zehu Yuan, Wanhong Li, Fadi Li, Xiangpeng Yue

**Affiliations:** 1 State Key Laboratory of Grassland Agro-Ecosystems; Key Laboratory of Grassland Livestock Industry Innovation, Ministry of Agriculture and Rural Affairs; College of Pastoral Agriculture Science and Technology, Lanzhou University, Lanzhou, 730020, P. R. China; 2 Engineering Laboratory of Sheep Breeding and Reproduction Biotechnology in Gansu Province, Minqin, 733300, P. R. China

## Abstract

Sheep milk is the most important feed resource for newborn lambs and an important food resource for humans. Sheep milk production and ingredients are influenced by genetic and environmental factors. In this study, we implemented selection signature analysis using Illumina Ovine SNP50 BeadChip data of 78 meat Lacaune and 103 milk Lacaune sheep, which have similar genetic backgrounds, from the Sheep HapMap project to identify candidate genes related to ovine milk traits. Since different methods can detect different variation types and complement each other, we used a haplotype-based method (hapFLK) to implement selection signature analysis. The results revealed six selection signature regions showing signs of being selected (P<0.001): chromosomes 1, 2, 3, 6, 13 and 18. In addition, 38 quantitative trait loci (QTLs) related to sheep milk performance were identified in selection signature regions, which contain 334 candidate genes. Of those, *SUCNR1* (succinate receptor 1) and *PPARGC1A* (PPARG coactivator 1 alpha) may be the most significant genes that affect sheep milking performance, which supply a significant indication for future studies to investigate candidate genes that play an important role in milk production and quality.

## 1 Introduction

Sheep have been raised for milk for thousands of years, which is much longer than cow milk production (Zervas and Tsiplakou, 2011). Sheep milk and its products are widely consumed in some parts of the world, especially in the Mediterranean. Sheep milk has a high degree of similarity to human milk in total fatty acid composition, which makes it a good raw material for infant formula production (Martin et al., 2016). In the sheep industry, prolific sheep usually cannot lactate enough milk for lambs, which could decrease lamb survival rate (Bradford, 1985). A milk replacer is sometimes used as alternative, but it is costly and labor-intensive. Therefore, it is important to identify genes related to sheep milk and then genetically improve sheep milk performance, which will obtain a better profitability in the sheep industry and diversify human milk resources.

Specialized strains of livestock have been cultivated by humans in long-standing husbandry practices; artificial and natural selection have imposed detectable selection signatures within genomes. These selection signatures can provide deep insights into selection mechanism and further uncover the causal genes related to relevant phenotypes. In sheep, selection signature analyses of closely related populations with divergent production purposes were successfully implemented in milk traits (Moioli et al., 2013), tail types (Moradi et al., 2012; Moioli et al., 2015; Yuan et al., 2017), gastrointestinal nematode-resistant traits (McRae et al., 2014) and reproductive traits (El-Halawany et al., 2016).

In order to find genes associated with ovine milk traits, researchers have conducted a selection signature analysis between five non-milk sheep breeds and five milk sheep breeds, and they have identified some milk-related genes such as *ABCG2* and *SPP1* (Gutierrez-Gil et al., 2014). However, this previous study still had some limitations in experimental design. First, the sheep breeds that they have chosen have different characteristics not only in milk performance but also in other phenotypic differences, such as in wool traits, which could lead to some false positives of the candidate genes. Meanwhile, this study analyzed milk Lacaune and meat Lacaune genome only using site frequency-based methods, although it has been suggested that the regression-based selection mapping approach is more accurate than that of haplotype-based analysis methods (e.g., extended haplotype homozygosity, EHH; integrated haplotype score, iSH) (Wiener and Pong-Wong, 2011). However, different methods can detect different variation types and complement each other, which could accurately and comprehensively reveal the selection signature that exists within the genome. With meat and milk Lacaune from the Sheep HapMap project (http://www.sheephapmap.org/hapmap.php, last access: 6 August 2019), we used a haplotype-based method (hapFLK) and considered population stratification to conduct the selection signatures searching within the sheep genome. We further identified genes related to sheep milk and then provided a potential theoretical basis for sheep breeding.

## 2 Materials and methods

### 2.1 Experimental data pre-processing

The Illumina Ovine SNP50 BeadChip data of 78 meat-purpose Lacaune sheep and 103 milk-purpose Lacaune sheep were downloaded from the Sheep HapMap project database (http://www.sheephapmap.org/, last access: 6 August 2019). The detailed description of these data was well done by Kijas et al. (2012). To facilitate subsequent gene annotation, the PLINK 1.07 software (Purcell et al., 2007) was used to upgrade the map file to match the sheep genome Oar_v3.1 and implement data quality control. Single-nucleotide polymorphisms (SNPs) were excluded from the subsequent analysis: (1) call rate < 90 %, (2) minor allele frequency (MAF) < 0.01 and (3) significantly deviated from Hardy–Weinberg equilibrium ($P<10^{-6}$).

### 2.2 Population structure analysis

The filtered SNPs were pruned using the indep-pairwise option (plink – file input – indep-pairwise 50 5 0.1) in PLINK 1.07 software (Purcell et al., 2007) to avoid the strong influence of SNP clusters in principal component analysis (PCA) and relatedness analysis. PCA identifies the principal components representing the population structure based on genetic correlations (shared identity-by-state segments) among individuals. The PCA was implemented using the snpStats R package (https://www.rdocumentation.org/packages/snpStats, last access: 6 August 2019). To verify PCA results, ADMIXTURE (Alexander et al., 2009) was implemented. ADMIXTURE estimates ancestry in a model-based manner from large autosomal SNP genotype datasets, and it includes a cross-validation procedure that allows the user to identify the number of presumed ancestral populations (K) for which the model has best predictive accuracy. In this study, K was set from 1 to 5, and a 10-fold cross-validation procedure was performed.

### 2.3 Genome scans for selection signatures using hapFLK

The hapFLK statistics detect selection signatures based on differences of haplotype frequencies between populations (Fariello et al., 2013). Considering the population stratification of samples, the hapFLK method, which is based on haplotype frequency and considers population stratification, was used in this experiment. We used hapFLK software to compute the hapFLK statistic and kinship matrix assuming 10 clusters in the fastPHASE model and used 98 Australia Poll Merino samples as an outgroup (http://www.sheephapmap.org/hapmap.php, last access: 6 August 2019). Then the hapFLK statistic was computed as the average across 20 expectation–maximization (EM) iterations to fit the LD model. However, the hapFLK statistic does not strictly follow any of the existing statistical distributions. To investigate the distribution of the hapFLK statistics, we plot a histogram of the hapFLK statistic. Then, we standardized hapFLK following Eq. (1):

1 $$\mathrm{Standardized}\;\mathrm{hapFLK}=\frac{\mathrm{Raw}\;\mathrm{hapFLK}-\mathrm{mean}(\mathrm{raw}\;\mathrm{hapFLK})}{\mathrm{SD}(\mathrm{raw}\;\mathrm{hapFLK})},$$

where SD (raw hapFLK) is standard deviation of raw hapFLK. Thus, the standardized hapFLK (Z scores) roughly follows a standardized normal distribution. Finally, we computed the P value for each SNP according to this standardized normal distribution.

### 2.4 Gene annotation

Candidate regions identified by hapFLK were annotated using ovine reference genome (Oar_v3.1). Gene function was annotated using the National Center for Biotechnology Information Gene (http://www.ncbi.nlm.nih.gov/gene/, last access: 6 August 2019), which was used for Gene Ontology (GO) analysis. The sheep QTL database (http://www.animalgenome.org/cgi-bin/QTLdb/OA/index, last access: 2 May 2019) was used to find whether the known milk-related QTLs are located in selection signature regions. In addition, the online database OMIM (http://www.ncbi.nlm.nih.gov/omim/, last access: 6 August 2019) and genomic information from other species, including humans, mice and bovines, were used to predict gene function.

### 2.5 GO and KEGG enrichment analysis

To extract biological meanings from the list of candidate genes, GO enrichment and Kyoto Encyclopedia of Genes and Genomes (KEGG) enrichment analyses were performed using the OmicShare tools (https://www.omicshare.com/tools/, last access: 6 August 2019). For GO enrichment, all candidate genes were mapped to GO terms in the Gene Ontology database. Gene numbers were calculated for every term, and significantly enriched GO terms in candidate genes compared to the genome background were defined by a hypergeometric test. The P value calculated from hypergeometric distribution follows Eq. (2):

2 $$P=1-\sum_{i=0}^{m-i}\frac{\left(\frac{M}{i}\right)\left(\frac{N-M}{n-i}\right)}{\frac{N}{n}},$$

where N is the number of all GO-annotated genes; n is the number of candidate genes in N; M is the number of particular GO-term-annotated genes in N; m is the number of particular GO-term-annotated genes identified by selection signature in M. For pathway enrichment analysis, significantly enriched pathways in candidate genes compared to the genome background were also defined by a hypergeometric test. The calculated P value went through fast discovery rate (FDR) correction following Eq. (3):

3 FDR=P⋅n/(rankP),

where P is the raw P value, n is the number of tests and rankP is the rank for the specific raw P value. Taking FDR ≤ 0.05, GO terms and pathways meeting this condition were defined as significantly enriched for candidate genes.

## 3 Results and discussions

### 3.1 Population genetic structure

**Figure 1:**
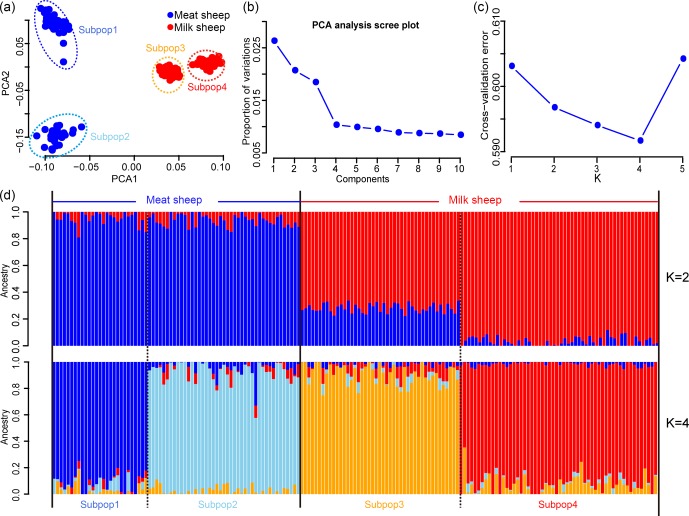
Population structure of the seven sheep populations. According to PC1, **(a)** all samples can be divided into four groups, and **(b)** the first two PCs can explain 2.8 % and 1.7 % variance respectively; **(c)** when K=4, the least amount of cross-validation error occurred; **(d)** makes it fairly clear that K=4 was the optimal modeling choice. The blue background represents the meat sheep group; the red background represents the milk group.

A total of 46 781 SNPs and 171 individuals were selected for further analysis after quality control. After implementing LD pruning, 13 935 SNPs with low LD were used for PCA and ADMIXTURE analysis. The PCA results showed that all animals can be divided into two groups by the first principal component (PC1): milk and non-milk sheep (Fig. 1a). The first two principal components (PC1 and PC2) can divide these samples into four subgroups (Fig. 1a) and explained 2.8 % and 1.7 % of the variance respectively (Fig. 1b). The results from the ADMIXTURE analysis showed that the least amount of cross-validation error occurred when K=4 (Fig. 1c) indicating that K=4 was the optimal modeling choice. Therefore, these samples could be appropriately divided into four subgroups (Fig. 1d), which was consistent with PCA.

### 3.2 Genome scans for selection using hapFLK

**Figure 2:**
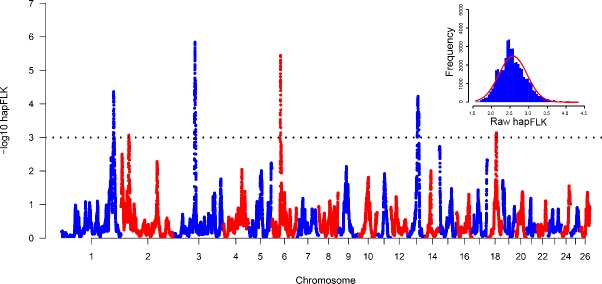
Manhattan plot of hapFLK statistics. The histogram in the top right shows the hapFLK statistics roughly followed a normal distribution. Black dotted line means the suggestive line; the -log⁡P value above this line means significant.

The hapFLK histogram shows that the hapFLK statistic approximately follows a normal distribution (Fig. 2, top right), which is similar to the previous study (Kijas, 2014; Yuan et al., 2017). Therefore, the P values could be calculated from the normal distribution. Negative ${\mathrm{log}}_{10}$ P values plotted in genomic order revealed six regions under strong selection (Fig. 2). The genomic location, size, peak SNP and peak genes in the selection regions identified using Oar_v3.1 (Jiang et al., 2014) were summarized in Table S1 in the Supplement. The average size of selective regions was 7.97 Mb ranging from 4.33 to 17.00 Mb. These six selected regions in this study were compared with the six convergence candidate regions (CCRs) identified by Gutierrez Gil et al. (2014). However, only an overlapping region (Chr6: 38.64–43.02 Mb) was found, and the majority of selection regions are not overlapping. A similar situation also appeared in the sheep-tail-type selection signature analyses (Moradi et al., 2012; Moioli et al., 2015). There are several reasons that may explain this result: (1) only one breed was analyzed in the current study, while 10 breeds were incorporated in a previous study (Gutierrez-Gil et al., 2014); (2) the methods between this study and Gutierrez-Gil et al. (2014) were different, which can detect different variants; (3) the low SNP density in both the current study and Gutierrez-Gil et al. (2014) might lead to a low statistical power (Simianer et al., 2014). These results suggested that separate populations selected for similar breeding goals have the low repeatability of selection signature analysis results.

### 3.3 Genes and functional annotations

To understand the function of these selection regions, we mapped them to the sheep genome Oar_v3.1 and sheep QTL database (http://www.animalgenome.org/cgi-bin/QTLdb/OA/index, last access: 2 May 2019). As a result, 38 QTLs related to milk traits (Table 1) and 334 candidate genes (Table S1) were identified in selected regions. These 38 milk-related QTLs were enriched in all milk-related QTLs in the sheep QTL database with a hypergeometric test of $P=2.65506\times 10^{-7}$ (significantly different from what is expected by chance). Significantly, the majority of milk-related QTLs (27 out of 38) were located at chromosome 1 (Chr1: 228.88–245.88 Mb). In detail, these 27 QTLs were linked to the percentage of milk fat and milk protein as well as yield of milk protein and milk fat in sheep (Sutera et al., 2019; Hao et al., 2019) suggesting that selection region on chromosome 1 may play an important role in sheep milk performance. In chromosome 13 selection region (Chr13: 43.57–53.55 Mb), two copy number variation regions (CNVRs) (Chr13: 48.83–49.71 Mb, Chr13: 49.01–49.71 Mb) were identified to be significantly ($P=6.051\times 10^{-7}$) associated with milk yield in Valle del Belice sheep (Di Gerlando et al., 2019). However, there was no known milk-related QTL in chromosome 18 selection region (Chr18: 37.95–42.60 Mb). This may be the relatively poor annotation of the current sheep QTL database (release 38), and this will be addressed when more milk-related QTLs are identified in the future.

**Table 1:** Milk-related QTLs located in selection regions.

Region	Chromosome	Start	End	Size	Sheep QTL
		(Mb)	(Mb)	(Mb)	
1	1	228.88	245.88	17.00	170 224 (Chr1: 233736829–233736869, MF) (Sutera et al., 2019)
					170 226 (Chr1: 233736829–233736869, PP) (Sutera et al., 2019)
					169 252 (Chr1: 236278074–236278174, PY) (Hao et al., 2019)
					169 251 (Chr1: 236278074–236278174, FY) (Hao et al., 2019)
					169 400 (Chr1: 236299569–236299669, FY) (Hao et al., 2019)
					169 150 (Chr1: 236964320–236964420, FY) (Hao et al., 2019)
					169 182 (Chr1: 237198394–237198494, FY) (Hao et al., 2019)
					169 181 (Chr1: 237198394–237198494, MY) (Hao et al., 2019)
					169 180 (Chr1: 237198394–237198494, PY) (Hao et al., 2019)
					169 524 (Chr1: 237368505–237368605, FY) (Hao et al., 2019)
					169 208 (Chr1: 237476668–237476768, MY) (Hao et al., 2019)
					169 207 (Chr1: 237476668–237476768, PY) (Hao et al., 2019)
					169 206 (Chr1: 237476668–237476768, FY) (Hao et al., 2019)
					169 442 (Chr1: 237702646–237702746, FY) (Hao et al., 2019)
					169 168 (Chr1: 237899096–237899196, MY) (Hao et al., 2019)
					169 167 (Chr1: 237899096–237899196, PY) (Hao et al., 2019)
					169 166 (Chr1: 237899096–237899196, FY) (Hao et al., 2019)
					169 446 (Chr1: 240960592–240960692, MY) (Hao et al., 2019)
					169 445 (Chr1: 240960592–240960692, FY) (Hao et al., 2019)
					169 280 (Chr1: 241703508–241703608, PY) (Hao et al., 2019)
					169 279 (Chr1: 241703508–241703608, FY) (Hao et al., 2019)
					169 499 (Chr1: 242397140–242397240, FY) (Hao et al., 2019)
					169 388 (Chr1: 242789285–242789385, FY) (Hao et al., 2019)
					169 387 (Chr1: 242789285–242789385, PY) (Hao et al., 2019)
					169 386 (Chr1: 242789285–242789385, MY) (Hao et al., 2019)
					169 551 (Chr1: 243458586–243458686, FY) (Hao et al., 2019)
					169 144 (Chr1: 243778419–243778519, FY) (Hao et al., 2019)
2	2	34.95	39.28	4.33	169 594 (Chr2: 37635669–37635769, MY) (Hao et al., 2019)
					13 911 (Chr2: 37102076–37260066, PP) (Gutierrez-Gil et al., 2009)
					57 738 (Chr2: 32023745–207420807, PP) (Garcia-Gamez et al., 2013)
					13 915 (Chr2: 8804882–248905321, MF) (Gutierrez-Gil et al., 2009)
3	3	91.37	97.96	6.59	57 740 (Chr3: 97143203–97187127, MY) (Garcia-Gamez et al., 2013)
4	6	38.64	43.94	5.30	169 477 (Chr6: 41850279–41850379, FY) (Hao et al., 2019)
					13 818 (Chr6: 43152047–43302377, MY) (Arnyasi et al., 2009)
					13 819 (Chr6: 43152047–43302377, MLACT) (Arnyasi et al., 2009)
					13 820 (Chr6: 43152047–43302377, MY) (Arnyasi et al., 2009)
					13 821 (Chr6: 43152047–43302377, MLACT) (Arnyasi et al., 2009)
5	13	43.57	53.55	9.98	169 479 (Chr13: 45264465–45264465, FY) (Hao et al., 2019)
6	18	37.95	42.60	4.65	–

Note: milk fat percentage – MF; milk protein percentage – PP; milk protein yield – PY; milk fat yield – FY; milk yield – MY; milk lactose yield – MLACT.

Of those candidate genes, succinate receptor 1 (Chr1: 234.11–234.16 Mb, *SUCNR1*) has been reported as close to the SNP rs417079368 (Chr1: 233.59 Mb), which was significantly ($P=4.07\times 10^{-7}$) associated with milk fat percentage and protein percentage in Valle del Belice sheep (Sutera et al., 2019). Its ligand, succinate, plays an important role not only in adenosine triphosphate generation (Littlewood-Evans et al., 2016) but also in signalling transduction by binding to and activating its specific receptor, SUCNR1 (also known as G-protein-coupled receptor-91, GPR91) (Mu et al., 2017). *SUCNR1* is expressed in multi-tissues and organs in sheep, such as the omentum, spleen, liver and mammary gland (Clark et al., 2017). Additionally, the expression of *SUCNR1* is related to milk protein trait in sheep (Suarez-Vega et al., 2016). *PPARGC1A* (Chr6: 43.23–43.33 Mb, PPARG coactivator 1 alpha) located near a multi-effect milk-related QTL region (Chr6: 43.15–43.30 Mb) (Arnyasi et al., 2009). Many scholars have shown that this gene was associated with milk production, milk fat percentage, and other milk-related properties (Khatib et al., 2007; Weikard et al., 2005; Schennink et al., 2009; Cong et al., 2016).

**Figure 3:**
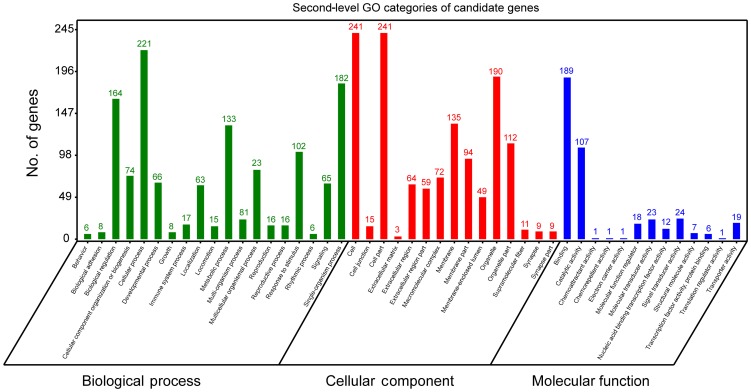
Second-level GO categories of candidate genes.

In order to further extract known biological meanings from these 334 candidate genes, GO and KEGG enrichment analyses were implemented using the OmicShare tools (https://www.omicshare.com/tools/, last access: 6 August 2019). GO analysis has shown that these candidate genes participated in 47 second-level GO categories (Fig. 3) and significant (FDR ≤ 0.05) enrichment in 12 GO terms (Table S1). The highest numbers of genes of second-level GO categories of biological processes (BPs), cellular components (CCs) and molecular function (MFs) are cellular process (GO: 0009987, biological process, 221 genes), cell (GO: 0005623, cellular components, 241 genes), cell part (GO: 0044464, cellular components, 241 genes) and binding (GO: 0005488, molecular function, 189 gene). Previously, genes involved in these second-level GO terms showed strong associations with ruminant milk productivities. For example, it has been reported that genes located in bovine milk yield QTL regions are preferred to enrichment in cellular process (GO: 000987), cell (GO: 0005623), cell part (GO: 0044464) and cellular process (GO: 0009987) (Salih and Adelson, 2009). Further, genes involved in cellular process (GO: 0009987) were found to be associated with fat yield, milk yield, protein yield and fertility index in Nordic red cattle (Iso-Touru et al., 2016). Also, miRNA target genes of goat mammary gland were enriched in cellular processes (GO: 000987, biological process), cell (GO: 0005623, cellular component), cell part (GO: 0044464, cellular component) and cellular process (GO: 0005488, molecular function) (Ji et al., 2012). All these findings support the candidate genes identified in the current study contribute to fundamental physiology of dairy sheep.

The most significant GO term of CC is cytoplasm (GO: 0005737, P=6.93E10-8, FDR = $2.98\times 10^{-5}$). The most significant GO term of MF was the G-protein coupled nucleotide receptor activity (GO: 0001608, $P=6.37007\times 10^{-5}$, FDR = $6.37007\times 10^{-5}$) and G-protein coupled purinergic nucleotide receptor activity (GO: 0045028, $P=6.37007\times 10^{-5}$, FDR = $6.37007\times 10^{-5}$). The finding that the cytoplasm GO term (GO: 0005737) was enriched in our gene set is interesting. Previous studies have reported that candidate genes associated with milk protein composition traits in a Chinese Holstein population were significantly (FDR = 0.0247) enriched in cytoplasm (GO: 0005737) (Zhou et al., 2019). Apart from GO analysis, KEGG analysis showed that candidate genes could be annotated to 36 KEGG classes (Fig. S1) and could participate in 173 pathways. The highest number of genes of KEGG categories was signal transduction (29 genes). However, no significant pathways were found.

## 4 Conclusions

Based on haplotype-based methods, the current study has six significant selection regions, which contains 38 known QTLs associated with milk yield. The identified six selection regions harbored 334 candidate genes. Some of the key candidate genes such as *SUCNR1* and *PPARGC1A* may play an important role in sheep milk performance. The findings from this study can be useful to optimize breeding programs to improve the milk-related traits after further functional studies and validation of the association in other independent populations.

## Supplement

10.5194/aab-62-501-2019-supplement The supplement related to this article is available online at: https://doi.org/10.5194/aab-62-501-2019-supplement.

## Data Availability

The data of the paper are available from Sheep HapMap project database (, International Sheep Genomics Consortium, 2019).
