# Supplementary material for: Selection signature analysis reveals genes underlying sheep milking performance
Source: Arch Anim Breed. 2019 Aug 8;62(2):501–8. doi: 10.5194/aab-62-501-2019 (PMC6859915; doi:10.5194/aab-62-501-2019)
Supplement: The supplement related to this article is available online at: https://doi.org/10.5194/aab-62-501-2019-supplement. [file aab-62-501-supplement.zip › aab-62-501-2019-supplement-title-page.pdf]

Supplement of Arch. Anim. Breed., 62, 501–508, 2019
https://doi.org/10.5194/aab-62-501-2019-supplement
© Author(s) 2019. This work is distributed under
the Creative Commons Attribution 4.0 License.

Open Access
Archives Animal Breeding

Supplement of

Selection signature analysis reveals genes underlying sheep milking performance

Zehu Yuan et al.

Correspondence to: Fadi Li (lifd@lzu.edu.cn) and Xiangpeng Yue (lexp@lzu.edu.cn)

- aab-62-501-2019-supplement-title-page.pdf
- AdditionalFiles
  - FigureS1.tif
  - Table S2 significant GO terms.xlsx
  - table S1.docx

The copyright of individual parts of the supplement might differ from the CC BY 4.0 License.
