# Supplementary material for: Selection signature analysis reveals genes underlying sheep milking performance
Source: Arch Anim Breed. 2019 Aug 8;62(2):501–8. doi: 10.5194/aab-62-501-2019 (PMC6859915; doi:10.5194/aab-62-501-2019)
Supplement: The supplement related to this article is available online at: https://doi.org/10.5194/aab-62-501-2019-supplement. [file aab-62-501-supplement.zip › AdditionalFiles/table S1.docx]

| Region | Peak SNP | Gene Number | Peak Gene | Genes |
| --- | --- | --- | --- | --- |
| 1 | OAR1_260750419.1 | 85 | *PHF20* | *DNAJA1*, *TIPARP*, *SSR3, KCNAB1, ODC1, DDX21, GMPS, SLC33A1, C3ORF33, PLCH1, RPL10, MME, ATAD1, GPR149, DHX36, ARHGEF26, YWHAH, THOC2, RAP2B, P2RY1, MBNL1, SUCNR1, AADAC, AADACL2, C1GALT1C1, UTP6, IGSF10, MED12L, P2RY12, P2RY14, GPR171, MED12L, CLRN1, FAM188B2, MANBA, SIAH2, FAM194A, SELT, EIF2A, TSC22D2, PFN2, RNF13, C3ORF16, COMMD2, WWTR1, TM4SF4, TM4SF1, TM4SF18, CP, HPS3, HLTF, GYG1, CPA3, CPB1, AGTR1, HMGB1, PA2G4, ZIC1, ZIC4, MAPRE1, PLSCR5, PLSCR2, PLOD2, PHF20, GLUD1, C3ORF58, SLC9A9, CHST2, U2SURP, PAQR9, PCOLCE2, TRPC1, PLS1, CCT2, ATR, XRN1, GK5, ATP1B3, ATP1B3, GRK7, RNF7, RASA2, ZBTB38, ACPL2, SPSB4* |
| 2 | OAR2_37472352.1 | 51 | *FRMD3* | *SLC28A3, RMI1, HNRNPK, KIF27, GKAP1, UBQLN1, C9ORF103, FRMD3, RASEF, C9ORF144B, CCL21, CCL19, CCL27, IL11RA, GALT, SIGMAR1, ARID3C, DCTN3, CNTFR, DNAI1, C9ORF25, KIAA1161, NUDT2, KIF24, UBAP1,* *RPL23, DCAF12, UBAP2, UBE2R2, NOL6, AQP7, NFX1, BAG1, CHMP5, SPINK4, B4GALT1, SMU1, DNAJA1, APTX, CLU, GULO, EPHX2, CHRNA2, PTK2B, TRIM35, STMN4, ADRA1A, DPYSL2, PNMA2, BNIP3L, PPP2R2A* |
| 3 | s60105.1 | 80 | *EXOC6B* | *BIRC6, YIPF4, NLRC4, SPAST, MEMO1, SRD5A2, XDH, FAM136A, STK38, RHOA, TGFA, ADD2, CLNS1A, CLEC4F, CD207, VAX2, ATP6V1B1, ANKRD53, TEX261, NAGK, PAIP2B, ZNF638, RPL21, DYSF, CYP26B1, EXOC6B, RPL10A, FTL, SPR, EMX1, SFXN5, RAB11FIP5, ACP1, SMYD5, PRADC1, CCT7, FBXO41, EGR4, ALMS1, NAT8B, TPRKB, DUSP11, DERA, C2ORF78, ACTG2, DGUOK, TET3, BOLA3, MOBKL1B, MTHFD2, SLC4A5, BCL2L1, DCTN1, RDH12, C2ORF81, WDR54, RTKN, INO80B, MOGS, MRPL53, CCDC142, LBX2, PCGF1, TLX2, DQX1,* *AUP1,* *HTRA2,* *LOXL3,* *DOK1,* *C2ORF65,* *SEMA4F,* *HK2,* *TACR1, EI24, FHL2, C2ORF49,* *TGFBRAP1, GPR45, MRPS9, GCSH* |
| 4 | OAR6_45004760.1 | 8 | *KCNIP4* | *SLIT2, PACRGL, KCNIP4, F52C9.6, RPL10A, GPR125, GBA3, PPARGC1A* |
| 5 | s60004.1 | 93 | *BMP2* | *KLF6, SCOC, PITRM1, PFK, ADARB2, F5, WDR37, IDI1, GTPBP4, LARP4B, DIP2C, CFDP2, ZMYND11, PRNP, RASSF2, SLC23A2, PCNA, CDS2, PROKR2, GPCPD1, CHGB, TRMT6, MCM8, CRLS1, LRRN4, FERMT1, BMP2, PPP1CC, MUTED, UBA52, HAO1, ADRA1D, SMOX, RNF24, PANK2, MAVS, C20ORF29, CDC25B, CENPB, SPEF1, HSPA12B, SIGLEC1, ADAM33, ATRN, WDR45L, C20ORF194, SLC4A11, ITPA, DDRGK1, PROSAPIP1, PROSAPIP1, FASTKD5, UBOX5, PTPRA, VPS16, FAM113A, C20ORF141, CPXM1, IDH3B, NOP56, TMC2, SNRPB, TGM6, TGM3, STK35, PDYN, SIRPA, SIRPA, FAM108C1, GINS1, PCMTD2, MYT1, NPBWR2, OPRL1, C20ORF201, RGS19, TCEA2, PRPF6, ZNF512B, DNAJC5, ZBTB46, ABHD16B, ZGPAT, ARFRP1, TNFRSF6B, RTEL1, STMN3, GMEB2, SRMS, PTK6, PPDPF, EEF1A2, KCNQ2* |
| 6 | s03048.1 | 17 | *ARHGAP5* | *TBA, FOXG1, PRKD1, RRP36, G2E3, SCFD1, COCH, STRN3, AP4S1, HECTD1, HEATR5A, C14ORF126, GPR33, NUBPL, ARHGAP5, AKAP6, NPAS3* |

Table S1 candidate genes harboured in six selection signature regions
