# Supplementary figures and images for: Selection signature analysis reveals genes underlying sheep milking performance
Source: Arch Anim Breed. 2019 Aug 8;62(2):501–8. doi: 10.5194/aab-62-501-2019 (PMC6859915; doi:10.5194/aab-62-501-2019)

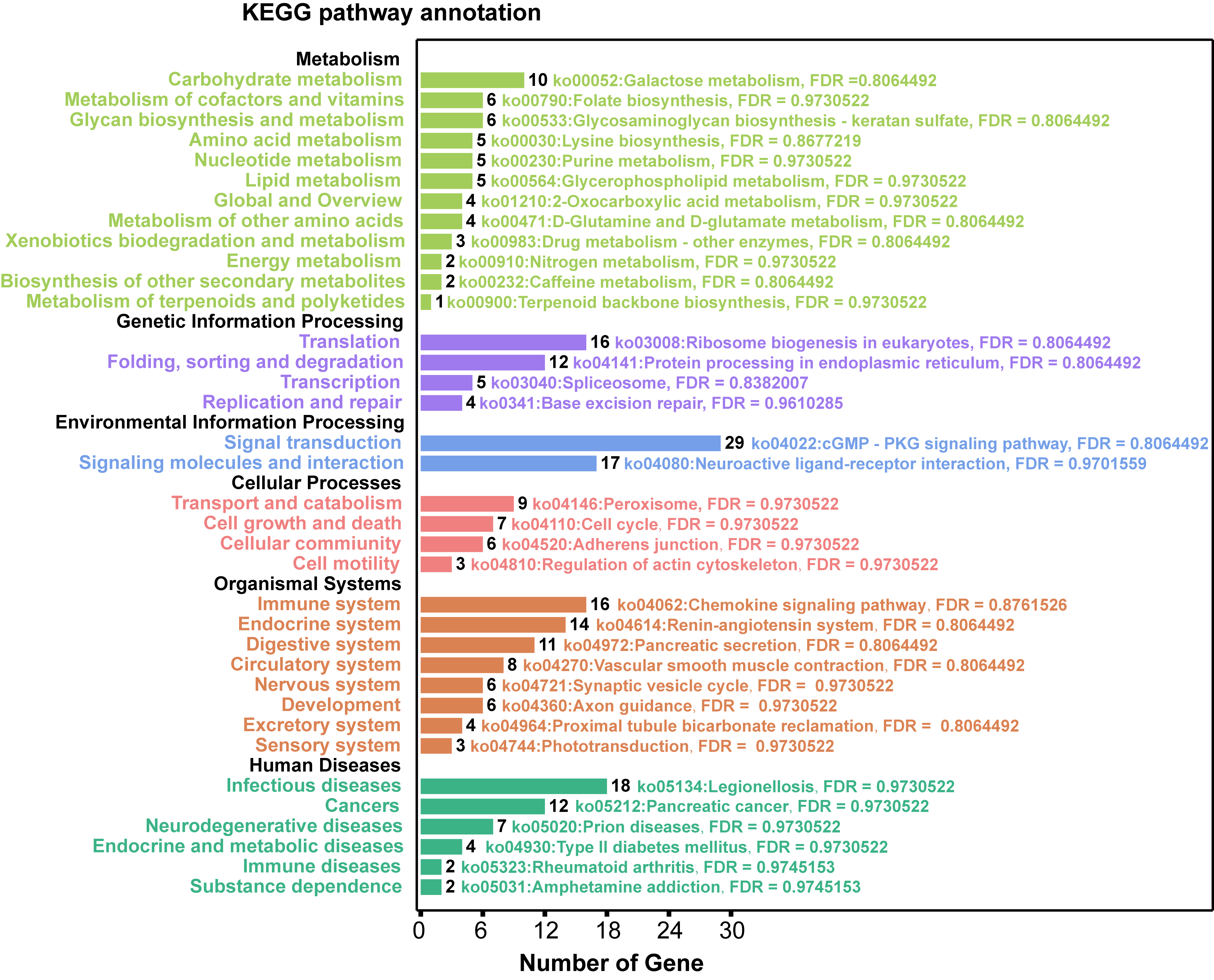

Supplement: The supplement related to this article is available online at: https://doi.org/10.5194/aab-62-501-2019-supplement. [file aab-62-501-supplement.zip › AdditionalFiles/FigureS1.tif]
